# Volar wrist ganglion associated with radial artery atherosclerosis: A case report

**DOI:** 10.1097/MD.0000000000034351

**Published:** 2023-07-14

**Authors:** Young-Keun Lee, Jong Hong Kim

**Affiliations:** a Department of Orthopedic Surgery, Research Institute of Clinical Medicine of Jeonbuk National University – Biomedical Research Institute of Jeonbuk National University Hospital, Jeonju, Republic of Korea.

**Keywords:** atherosclerosis, ganglion, radial artery, wrist

## Abstract

There have been no reports of volar wrist ganglions being associated with atherosclerosis of the radial artery. Good results were obtained with radial artery reconstruction through ganglion excision and autogenous vein grafting. Hence, we report a previously unreported case, along with a review of the literature.

**Patient concerns:**

A 58-year-old female presented with the chief complaint of a mass on the volo-radial side of her right wrist. The patient complained of a tingling sensation in the thumb, index, and extensor zones that worsened when pressing the mass.

**Diagnoses:**

Sonography revealed a well-defined, anechoic cystic lesion adjacent to the radial artery.

**Interventions:**

Exploration was performed using a zig-zag incision on the mass. The superficial radial nerve (SRN), which innervates the thumb, was distorted by the mass and the nerve dissected from the mass. However, the artery and ganglion cysts were not separated completely in a part where hardening of the artery wall progressed as a result of degenerative changes, showing multiple small, hard, and yellowish masses. We resected the radial artery (approximately 1.5 cm) along with the ganglion and sent it for histological examination. The radial artery was then reconstructed using an autogenous venous graft.

**Outcomes:**

At the 34-month follow-up, the patient was asymptomatic. Radial artery patency was normal without recurrence of the ganglion cyst.

**Lessons:**

In patients with risk factors for radial artery atherosclerosis, a more careful diagnosis is required for the surgical treatment of the volar wrist ganglion. In addition, if the ganglion and radial artery are not completely dissected, excision of the radial artery and subsequent reconstruction of the radial artery using an autogenous vein may be a good surgical strategy.

## 1. Introduction

The volar wrist ganglion is the second most common ganglion in the hand and wrist regions, accounting for 18% to 20% of cases.^[1]^ Ganglion cysts are mostly asymptomatic, with the exception of swelling. The treatment options are variable and include reassurance, nonsurgical methods such as aspiration with or without steroid injections or hyaluronidase, and surgical excision.^[2]^ Among these, surgery is associated with a lower recurrence rate than conservative treatment. However, it has a higher rate of complications and longer recovery period. The rate of symptomatic relief might not be higher than that of the other treatments.^[3]^ Nevertheless, in cases where the patient wishes to remove the lesion for esthetic purposes or where pain and/or nervous symptoms are induced, surgical excision should be considered.^[2–4]^

Some reports have indicated that the volar wrist ganglia convey a worse prognosis, with a higher complication rate than the dorsal ganglion.^[1,5]^ This is attributed to a number of factors, such as the close proximity of the volar ganglia to important anatomic structures such as the radial artery, palmar cutaneous branch of the median nerve, and the number of different origins of the ganglion.^[1,5]^ Therefore, surgical treatment of the volar ganglion should be performed carefully by an experienced senior surgeon.

Despite these possible complications and the poor outcomes of surgical treatment, we obtained a satisfactory outcome using a surgical approach in a case of volar wrist ganglion associated with radial artery atherosclerosis. Hence, we report a previously unreported case along with a review of the literature.

### 1.1. Consent

The patient signed an informed consent form for the publication of this case report and any accompanying images. Ethical approval for this study was waived by the ethics committee of Jeonbuk National University Hospital because it was a case report and there were fewer than 3 patients (December 25, 2022).

## 2. Case presentation

A 58-year-old female presented with the chief complaint of a mass on the volo-radial side of her right wrist. The lesion had emerged 2 years previously. It has expanded over the past 5 months. No specific treatments were administered. The patient was right-handed. She has been employed in sewing for 8 hours a day for 5 or more days a week over the past 30 years.

The patient had undergone total thyroidectomy for thyroid cancer at another hospital 30 years previously, for which she had been taking medication. In addition, the patient was taking medication for hypertension, which had been diagnosed 25 years previously at another hospital. There had no history of wrist injury or surgery.

Physical examination revealed a 3 × 3 cm, non-tender, soft mass on the volo-radial side of the right wrist. The patient complained of a tingling sensation in the thumb, index, and extensor zones that worsened when pressing the mass. The range of motion of the right wrist was 204°, which is the same as that of the left wrist. It did not induce pain.

**Figure 1.**
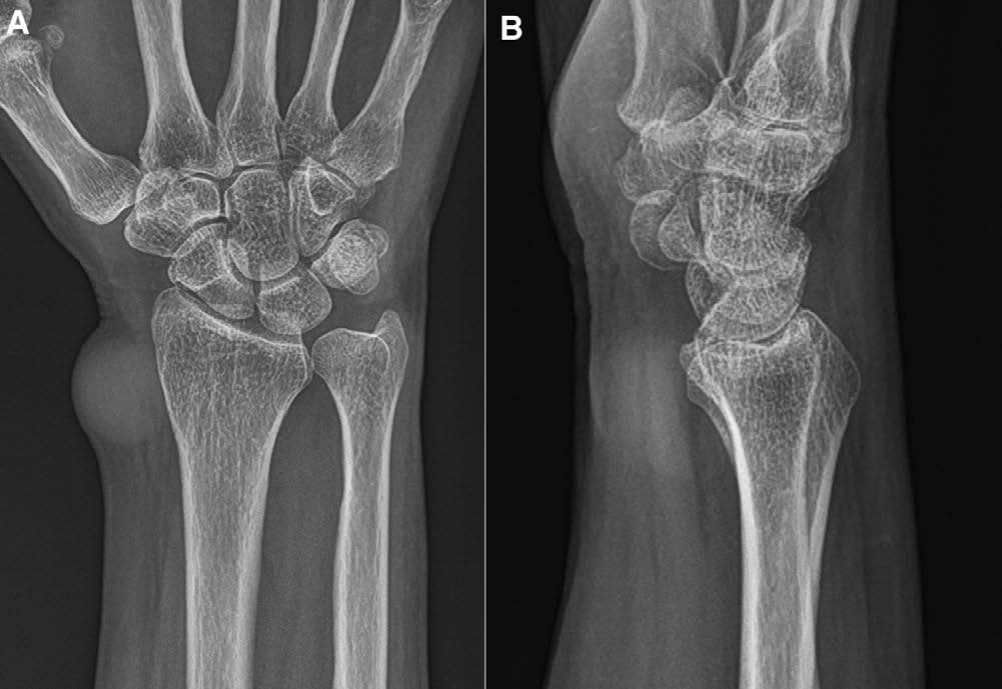
Initial plain anteroposterior (A) and lateral (B) radiographs of the right wrist showing an increased fort tissue contract suspicious for a mass lesion in the radial and volar sides of the radial styloid process without other findings of arthritis.

**Figure 2.**
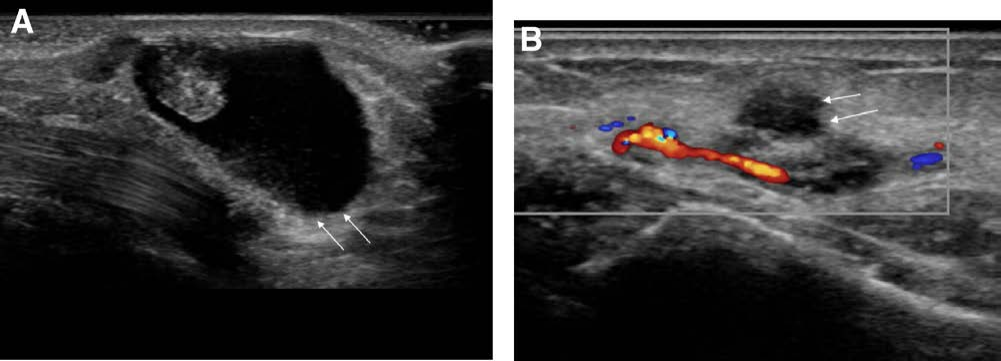
(A and B) Sagittal sonography show a well-defined anechoic ganglion cyst (arrows) connected with radial artery.

Plain radiography revealed an increased soft tissue shadow on the radial and volar sides of the radial styloid process, which was suspected to be a mass. No other signs of arthritis (Fig. 1A and B). Sonography revealed a well-defined, anechoic cystic lesion adjacent to the radial artery (Fig. 2). As the patient wanted to remove the lesion and complained of irritation symptoms in the superficial radial nerve (SRN), we decided to perform surgical treatment.

**Figure 3.**
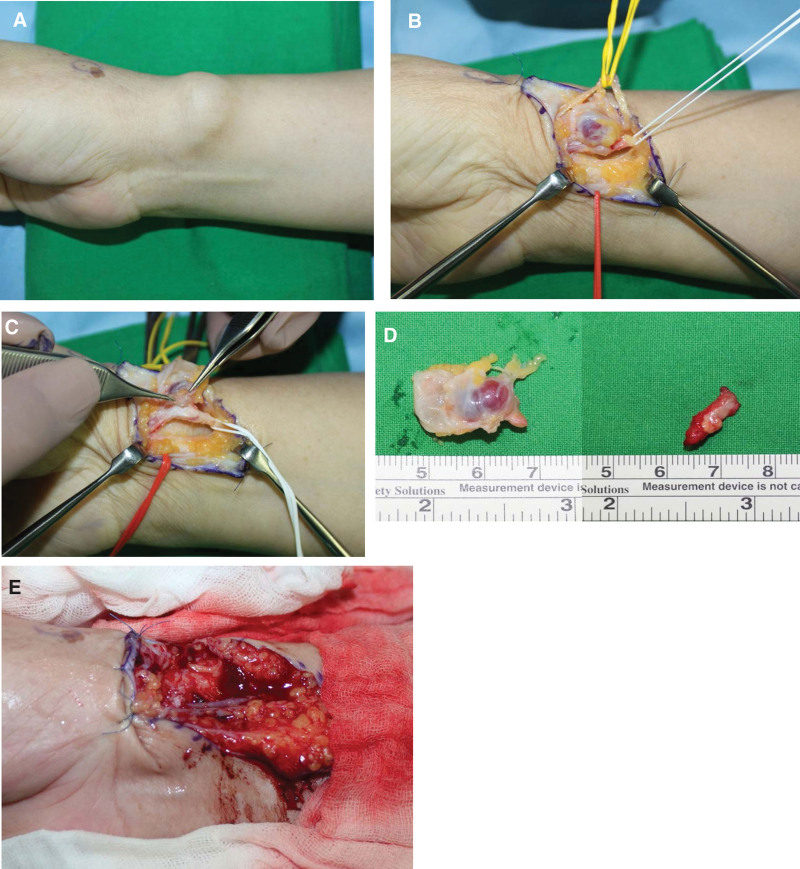
Intraoperative photographs. (A) A 3 × 3 cm sized ganglion cyst in the volo-radial aspect of the right wrist. (B) The SRN dissected from the mass. (C) Ganglion cyst and the radial artery were not completely separated, and the radial artery wall hardened, contained small sized hard yellowish masses. (D) An excised ganglion cyst together with a radial artery segment. (E) Radial artery was reconstructed with autogenous vein graft (2 cm) from ipsilateral forearm.

**Figure 4.**
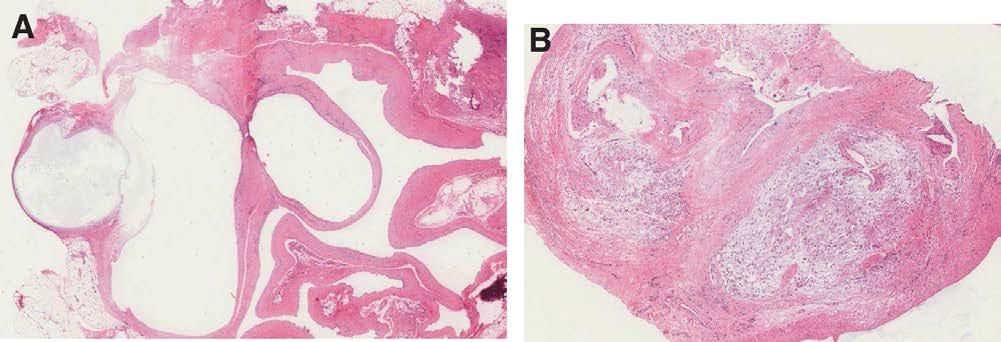
Microscopic appearance of the excised mass (A) and radial artery (B) showing fibrotic connective tissue without epithelial cell lining or atherosclerosis (hematoxylin-eosin, original magnification ×10, 40, respectively).

The operation was performed under regional anesthesia using a tourniquet. Exploration was performed using a zig-zag incision in the mass (Fig. 3A). As the SRN, which innervates the thumb, was distorted by the mass, the nerve was dissected from the mass (Fig. 3B). Subsequently, the radial artery is dissected from the mass. However, the artery and ganglion cysts were not separated completely in the part where hardening of the artery wall progressed as a result of degenerative changes, showing multiple small, hard yellowish masses (Fig. 3C). There was no communication between the wrist joint and the ganglion. Considering the risk of recurrent ganglion cysts associated with incomplete resection, approximately 1.5 cm of the degenerative radial artery was resected along the ganglion (Fig. 3D). The radial artery was reconstructed by vein grafting using an approximately 2 cm vein taken from the forearm (Fig. 3E). The resected ganglions and radial arteries were sent for histological examination. The final histopathological diagnosis of the mass was ganglion cyst, and atherosclerosis of the radial artery was identified (Fig. 4).

After surgery, the right wrist was immobilized with an above-elbow plaster splint for 2 weeks. A below-elbow plaster was then applied for an additional 2 weeks. Unrestricted full active motion was permitted at 4 weeks.

**Figure 5.**
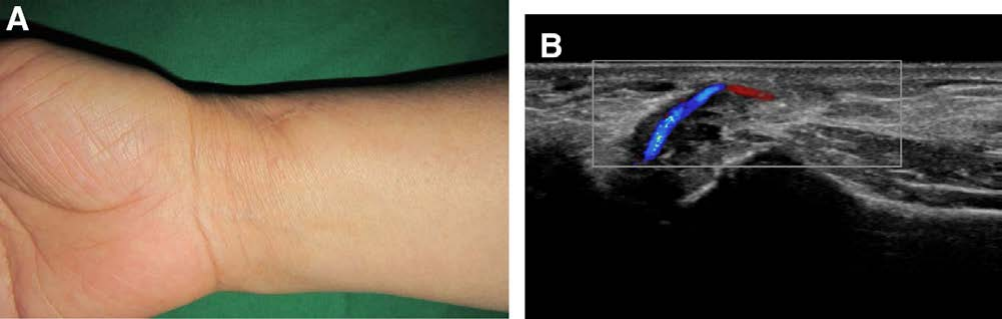
Follow-up photographs (A) and sonograph (B) obtained at 34 months post operation revealing no recurrence of ganglion cyst with patency of the radial artery maintained (note continuity of the radial artery).

The patient was asymptomatic during the 34-month follow-up visit. The patient exhibited a normal range of motion in the right wrist. Radial artery patency was normal without recurrence of the ganglion cyst (Fig. 5).

## 3. Discussion

Volar wrist ganglion occurs relatively commonly, accounting for 18% to 20% of all ganglions arising in the hand and wrist.^[1]^ No treatment, except follow-up, was required in most cases. However, treatment is recommended in cases of esthetic problems, pain, and/or tingling symptoms. To date, open resection is the treatment of choice. However, Mathoulin et al^[6]^ recommended arthroscopic treatment. After experiencing 2 cases of radial artery injury in the early years using an arthroscope for ganglion removal, we are not currently performing arthroscopic excisions.

The volar side of the wrist is closely associated with the main anatomical structures, such as the radial artery and the SRN. Ganglions in this region have high recurrence rates 20% to 42%.^[1,7]^ Although the exact cause of this recurrence remains unclear, inadequate and incomplete excision is one of its main reasons.^[1,4]^ We suspect that passive treatment to avoid injury to the main anatomical structures may have led to inadequate and incomplete excisions. To reduce the recurrence rate, Athanasian and Puhaindran recommended that the joint should be opened and explored. Thereafter, approximately 3 ± 4 mm of the capsular attachments were removed because the ganglion pedicle extended to the volar joint capsule.^[4]^ Jacobs and Govaers^[1]^ reported a recurrence rate of 28% and an injury rate of 28% in the palmar cutaneous branch of the median nerve during volar ganglion excision, highlighting the advantages of surgery performed by an experienced surgeon to reduce the recurrence rate and complications.

The radial artery in the wrist is commonly affected by atherosclerosis owing to its vasoreactivity and anatomical characteristics, particularly in the elderly population.^[8,9]^ Risk factors for radial artery atherosclerosis include age (≥50 years), smoking, diabetes, and peripheral vascular disease.^[10]^ In the present case, the patient’s age was the only risk factor associated with atherosclerosis. However, treatment for hypertension over the past 25 years may have been a predisposing factor. To date, there have been no reports of wrist ganglions being associated with radial artery atherosclerosis.

Although no communication was observed intraoperatively between the ganglion and the wrist joint, it was difficult to completely dissect the radial artery. Therefore, this study aimed to develop an alternative surgical approach. Sawyer et al^[11]^ reported that complications could be prevented by not removing the surface in contact with the radial artery. However, we speculated that the remaining area adjacent to the radial artery with no communication with the joint might be the cause of ganglion recurrence. Therefore, we decided to resect the radial artery and reconstruct it by using an autogenous vein obtained from the forearm.

The radial artery is commonly affected by atherosclerosis. Therefore, we believe that atherosclerosis can cause volar wrist ganglion recurrence after surgical treatment in patients with risk factors for the disease. Further studies on the relationship between atherosclerosis and the ganglion are needed for careful and active diagnosis.

This study is limited in that it reports a single case, which precludes comparison with a case that prevented the radial artery from being affected by atherosclerosis. Consequently, it was difficult to determine whether our surgical approach was the best strategy.

## 4. Conclusion

Atherosclerosis should be identified through careful diagnosis to achieve successful surgical treatment of volar wrist ganglion in patients with risk factors for radial artery atherosclerosis. In addition, if the ganglion and radial artery are not completely dissected, excision of the radial artery and subsequent reconstruction of the radial artery using an autogenous vein might be a good surgical strategy.

## Author contributions

**Conceptualization:** Young-Keun Lee.

**Data curation:** Young-Keun Lee.

**Formal analysis:** Young-Keun Lee, Jong Hong Kim.

**Supervision:** Young-Keun Lee.

**Writing – original draft:** Young-Keun Lee, Jong Hong Kim.

**Writing – review & editing:** Young-Keun Lee, Jong Hong Kim.
